# Identifying Patients With Asymptomatic Hyperparathyroidism by Serum Calcium and Vitamin D Screening in West Bengal, India

**DOI:** 10.7759/cureus.81869

**Published:** 2025-04-08

**Authors:** Antarip Bhattacharya, Dhritiman Maitra, Uttam Mondal

**Affiliations:** 1 General Surgery, Newham University Hospital, Barts Health National Health Service Trust, London, GBR; 2 Breast and Endocrine Surgery, Medical College Kolkata, Kolkata, IND; 3 General Surgery, Medical College Kolkata, Kolkata, IND

**Keywords:** asymptomatic hyperparathyroidism, hypercalcemia, primary hyperparathyroidism, screening, vitamin d deficiency

## Abstract

Background: Primary hyperparathyroidism (PHPT) is the leading cause of hypercalcemia, with solitary parathyroid adenoma responsible for most cases. Many patients with hyperparathyroidism remain asymptomatic, but early detection and parathyroidectomy can prevent complications such as nephrolithiasis, pancreatitis, and bone and kidney disorders. This study emphasizes the importance of screening for asymptomatic hyperparathyroidism to enable timely intervention.

Methods: Serum calcium and Vitamin D3 levels were screened in 6000 outpatients between March 2021 and September 2022. Patients with known hyperparathyroidism, conditions altering calcium metabolism, or those on calcium/Vitamin D supplements were excluded. Vitamin D deficiency was corrected in normocalcemic patients, and follow-up calcium levels were measured. Hypercalcaemic cases underwent additional phosphate and intact parathormone (iPTH) testing. Hyperparathyroidism was confirmed using standard diagnostic criteria, followed by gland localisation and surgery.

Results: Among 6000 patients screened, 85.2% (5112) had normocalcemia with Vitamin D deficiency. Hypercalcemia was initially observed in three patients, with two additional cases identified post-Vitamin D correction. Of the five hypercalcaemic patients, three were confirmed to have high iPTH levels and low phosphate, leading to surgical intervention. Asymptomatic hyperparathyroidism prevalence was 0.05% (3/6000), constituting 15.8% of all PHPT cases encountered during the study period.

Conclusion: While global asymptomatic hyperparathyroidism prevalence is approximately 1%, this study observed a lower incidence (0.05%) in a West Bengal population. Despite this, early screening and intervention are vital to prevent long-term complications and reduce healthcare burdens.

## Introduction

Primary hyperparathyroidism, the leading cause of hypercalcemia [1,2], results from the autonomous production of parathormone (PTH) and ranks as the third most prevalent endocrine disorder [3]. This condition affects up to 2% of postmenopausal women [4,5] and is marked by hypercalcemia paired with elevated or inadequately suppressed levels of parathyroid hormone, primarily due to a solitary parathyroid adenoma in 85-90% of cases [6,7]. Associated health issues and expensive complications include reduced bone mineral density, fractures, and kidney stones [8,9].

Hypercalcemia, and consequently primary hyperparathyroidism (PHPT), are more prevalent than previously thought. This has led to PHPT being recognized as a common endocrine disorder. PHPT is a chronic condition with a range of clinical manifestations. Very few cases are truly 'asymptomatic,' meaning no symptoms or organ issues are detected even after thorough screening [10].

Many patients exhibit 'classically symptomatic' PHPT, presenting with symptoms like bone pain, kidney stones, peptic ulcers, pancreatitis, and hypercalcaemic crises [11]. Today, due to routine calcium screening, most patients fall under the 'minimally symptomatic' category. These individuals experience symptoms associated with hypercalcemia, such as depression, lethargy, cognitive changes, insomnia, headaches, muscle weakness, frequent urination, constipation, weight loss, nausea, and overall reduced quality of life.

The distinction between 'mild' and 'asymptomatic' PHPT is not clearly defined in the literature. Thus, recent international consensus often treats 'asymptomatic' and 'mildly symptomatic' PHPT as the same [12,13]. 'Mild' or 'asymptomatic' PHPT involves abnormal parathyroid gland activity, as indicated by lab results, without clear symptoms of excessive calcium or parathyroid hormone, and without formal surgical indications. Familial hypocalciuric hypercalcemia is excluded from this definition. Risk factors for PHPT are not well understood [14]. Monogenic disorders like multiple endocrine neoplasia types I and II and neck irradiation account for less than 5% of cases [15,16].

Before the 1960s, PHPT was rare, with patients showing severe symptoms such as osteitis fibrosa cystica and kidney stones. The advent of automated serum calcium measurement in the 1970s allowed for earlier diagnosis through incidental hypercalcemia findings [17]. PHPT now affects about 1% of the general adult population and 2% of postmenopausal women [18-20]. In Western countries, only a small percentage of PHPT patients present with severe symptoms like nephrolithiasis and osteitis fibrosa cystica. Over 80% of patients do not exhibit the classic severe symptoms [21-24].

The presentation of PHPT varies globally. In India, most patients have severe symptoms, with only 5% being asymptomatic. Many have significant bone disease or kidney stones [25]. The situation in other developing regions is likely similar. The shift in clinical presentation in the Western world has led to questions about the appropriateness of traditional surgical management for PHPT [26]. Guidelines recommend surgery for patients with clear symptoms, while those without symptoms but with markers of severe or progressing disease are also advised to consider surgery. Otherwise, observation is recommended, especially if the patient cannot adhere to medical surveillance.

PHPT is now recognized as relatively common, with an incidence rate of 22 cases per 100,000 people per year. Studies indicate that about 2% of postmenopausal women have PHPT, peaking in the seventh decade of life, with women constituting three-quarters of cases [27,28]. PHPT and malignancy account for 90% of hypercalcemia cases, with PHPT being the leading cause in outpatients and malignancy in hospitalized patients [29]. Hypercalcemia due to malignancy includes humoral hypercalcemia of malignancy, hypercalcemia from bone metastases, and hematologic malignancies like multiple myeloma, often involving parathyroid hormone-related peptide (PTHrP) [30-32].

Most currently diagnosed PHPT patients are mild or asymptomatic, particularly postmenopausal women. Parathyroidectomy can confirm the diagnosis and alleviate discomfort, prevent complications, and offer financial benefits. However, many mild or asymptomatic cases remain stable, with few at risk of complications. Regular physical exercise, proper diet, adequate calcium and water intake, and correction of Vitamin D deficiency are recommended. Both surgeons and medical practitioners agree that medical treatments like calcimimetics and bisphosphonates are unnecessary for mild or asymptomatic cases. Further studies are needed to understand the long-term outcomes of treated and untreated PHPT in terms of fractures, cardiovascular risk, and mortality, as well as the cost/benefit ratio of surgery versus observation [33].

In detecting patients with asymptomatic hyperparathyroidism, we can provide them with an opportunity to get operated on at an early stage and prevent hyperparathyroidism-related complications in later life. Importantly, such early identification and intervention may lead to improved long-term outcomes by reducing the risk of nephrolithiasis, osteoporosis-related fractures, and cardiovascular morbidity. Furthermore, it can help lower the healthcare burden associated with delayed diagnosis and advanced disease presentations in resource-limited settings.

## Materials and methods

Aims and objectives

The primary aim of this study was to detect asymptomatic hyperparathyroidism among patients attending the General Surgery Outpatient Department at Medical College, Kolkata. This objective was pursued through the screening of serum calcium and Vitamin D3 levels, recognizing that early detection of asymptomatic hyperparathyroidism could prevent the progression to symptomatic disease, thereby reducing the morbidity associated with untreated hyperparathyroidism. The secondary objectives of the study were twofold: firstly, to identify and correct hypocalcaemia arising from Vitamin D deficiency, and secondly, to facilitate early surgical intervention for those diagnosed with asymptomatic hyperparathyroidism to preempt the onset of disease-related complications.

This study adopted an observational design and was situated in the Outpatient Department of the Department of General Surgery at Medical College, Kolkata, a prominent tertiary multi-disciplinary medical institution. The research was conducted over an 18-month period, reflective of the need for a comprehensive timeframe to accrue a sufficiently large sample for robust analysis.

Methods

The participant pool consisted of patients attending the General Surgery Outpatient Department who consented to join the study after satisfying the eligibility criteria. To capture a representative sample of the outpatient population, the study included a wide age range of participants from 20 to 60 years, excluding those with known conditions that could skew serum calcium and Vitamin D measurements, such as chronic kidney disease, bone diseases, or a history of carcinoma. Additionally, individuals currently on calcium or Vitamin D supplementation, undergoing hemodialysis, or with a history of hypercalcemia-related diseases like tuberculosis or sarcoidosis were excluded to mitigate confounding factors.

A total of 6000 patients were strategically selected to ensure the study was powered adequately to detect even a low prevalence of asymptomatic hyperparathyroidism. Inclusion and exclusion criteria were rigorously applied to refine the study cohort, enhancing the validity of the screening outcomes.

**Figure 1:**
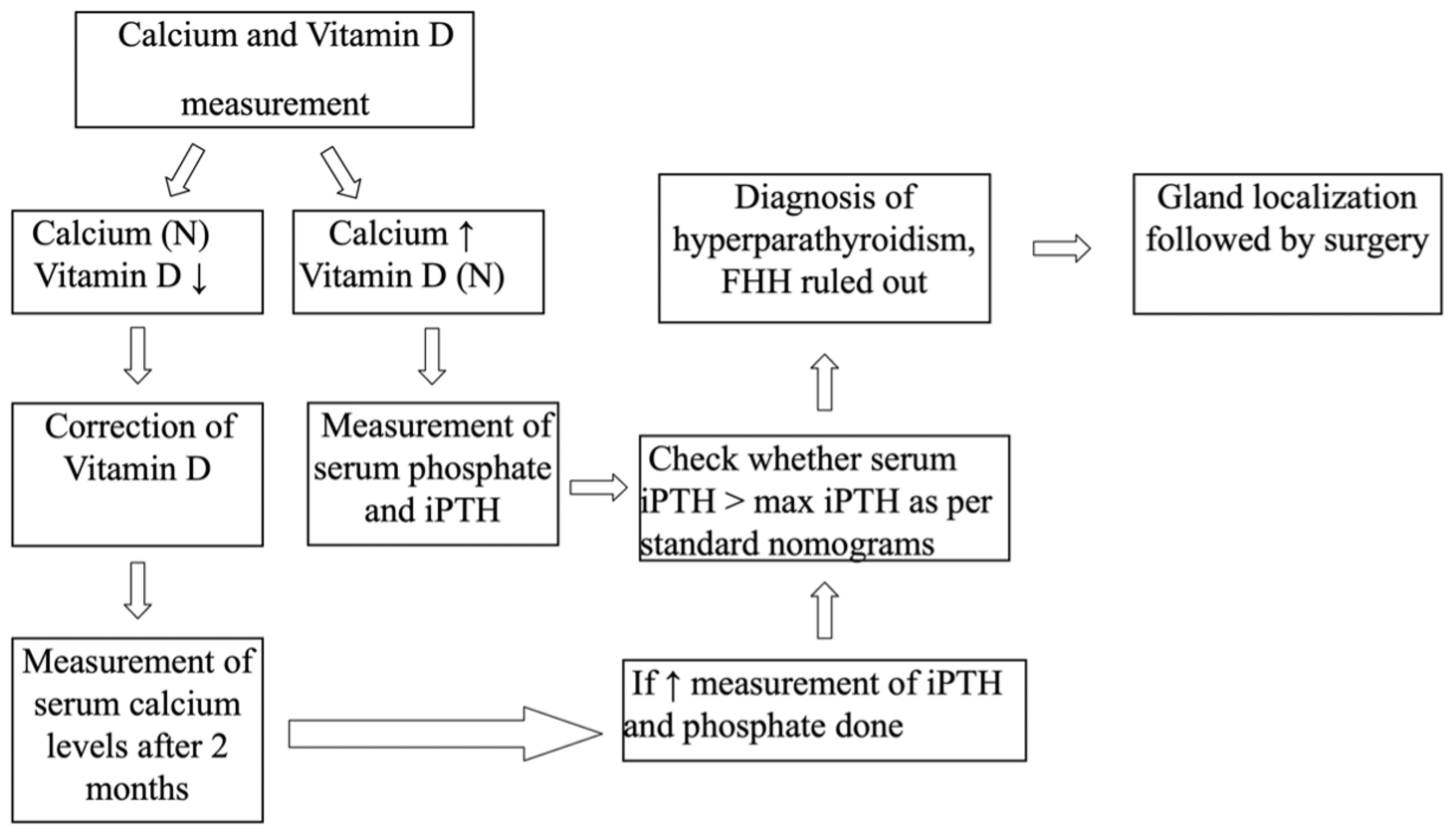
Flow chart showing the work plan FHH: Familial hypocalciuric hypercalcaemia; iPTH: Intact parathyroid hormone.

Key study variables included demographic information (age and sex) and biochemical markers (serum calcium and Vitamin D levels). Each participant underwent a detailed screening process where Vitamin D deficiency was corrected where necessary, and follow-up serum calcium levels were measured to ascertain any emergent hypercalcaemia post-correction. Patients identified with low serum Vitamin D3 levels (below 30 ng/ml) were administered cholecalciferol (Vitamin D3) supplementation. The correction protocol consisted of oral cholecalciferol 60,000 IU once weekly for eight weeks, followed by a maintenance dose of 60,000 IU monthly. Compliance was verbally confirmed at follow-up visits. Serum calcium levels were reassessed eight weeks after initiating supplementation to evaluate for unmasking of hypercalcemia. The exact pathway is described in Figure 1.

Statistical analysis

Statistical analysis was performed using standard software packages recognized for medical research. Descriptive statistics were first utilized to summarize the basic features of the data, such as mean, standard deviation, and range for continuous variables, and frequencies and percentages for categorical variables. To test for significant differences in serum calcium levels before and after Vitamin D correction, paired sample t-tests were conducted. The association between demographic variables (such as age and sex) and hypercalcemia incidence was assessed using the Chi-square test for categorical variables and independent sample t-tests for continuous variables.

Furthermore, logistic regression analyses were employed to examine the impact of Vitamin D correction on the likelihood of developing hypercalcemia, adjusting for potential confounders identified during the initial analysis phase. This multivariable approach allowed for the assessment of independent factors contributing to the primary outcome, providing insights into the complex interplay of biochemical markers and demographic characteristics.

Ethical approval

This study was reviewed and approved by the Institutional Ethics Committee of Medical College, Kolkata, under the registration number ECR/287/Inst/WB/2013/RR-19. The approval was granted on January 27th, 2021, with Ref No. MC/KOL/IEC/NON-SPON/991/01/2021 . Ethical conduct of the study was overseen in accordance with the ethical standards of the National Research Committee and with the 1964 Helsinki Declaration and its later amendments. The committee convened in the Council room at Medical College, Kolkata, and included members from various departments, ensuring a comprehensive review of the study's adherence to ethical guidelines. Approval was given to proceed with the study as proposed without modifications, affirming compliance with both the Indian Council of Medical Research (ICMR) and New Clinical Trial Rules 2019 guidelines. All participants in this study provided informed consent, and all procedures followed were in accordance with the ethical standards of the responsible committee on human experimentation.

## Results

Our study's comprehensive screening of 6000 patients at the Outpatient Department of the Medical College, Kolkata, offers unique insights into the prevalence and characteristics of asymptomatic hyperparathyroidism in West Bengal, India.

Demographic and clinical characteristics

**Figure 2:**
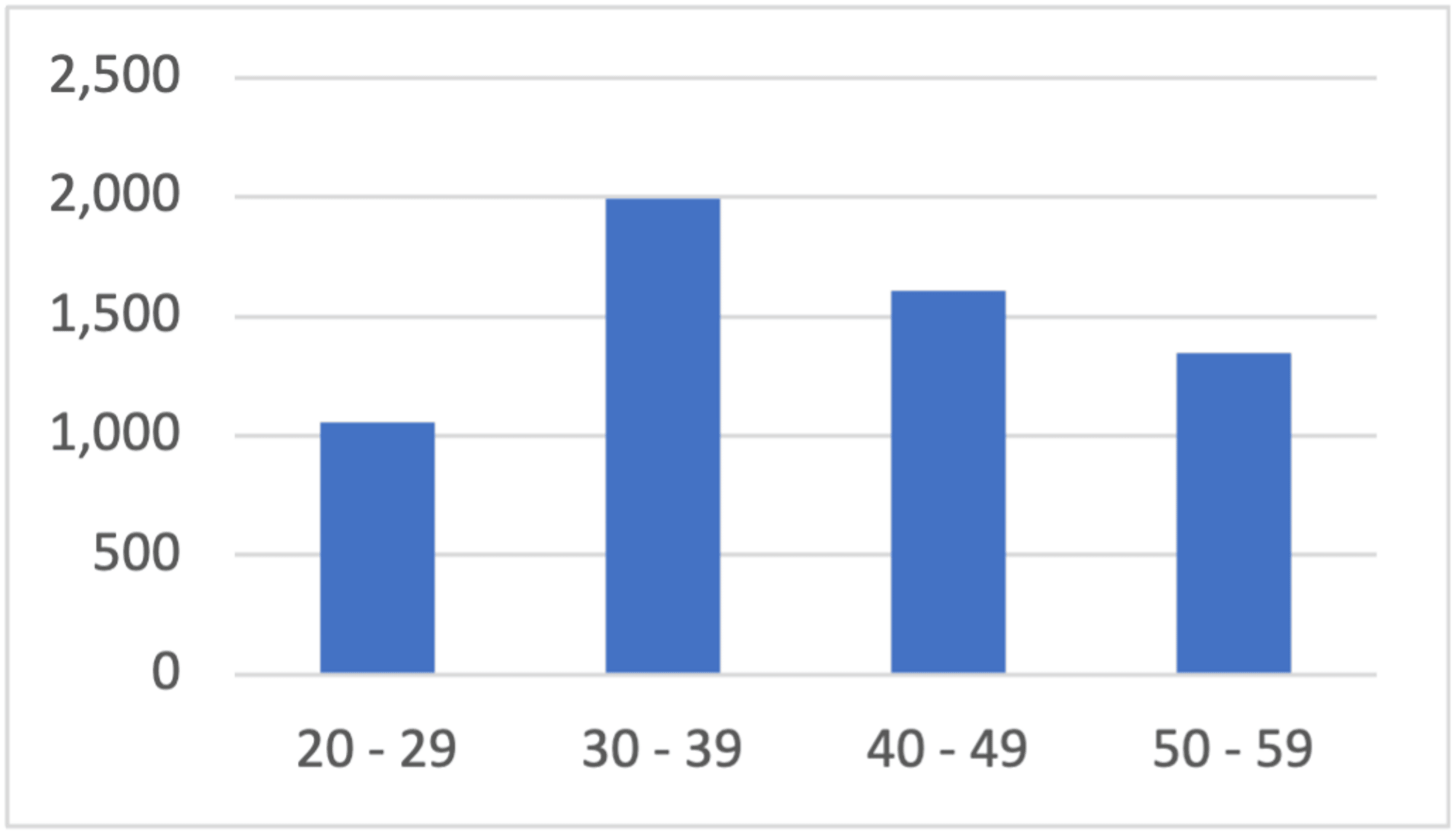
Bar diagram showing distribution of age of study population X-axis: Age in Years; Y-axis: Number of subjects in the study populatiopn.

The demographic analysis indicated a predominant age group of 30 to 39 years, representing 33.2% of the study population, as detailed in Table 1. The distribution across different age groups is visually represented in Figure 2, a bar diagram highlighting the concentration of cases in the middle-aged population.

**Table 1:** Age distribution of the study population

Age Group	Number	Percentage (%)
20 - 29	1,056	17.6
30 - 39	1,992	33.2
40 - 49	1,608	26.8
50 - 59	1,344	22.4
Total	6,000	100

**Figure 3:**
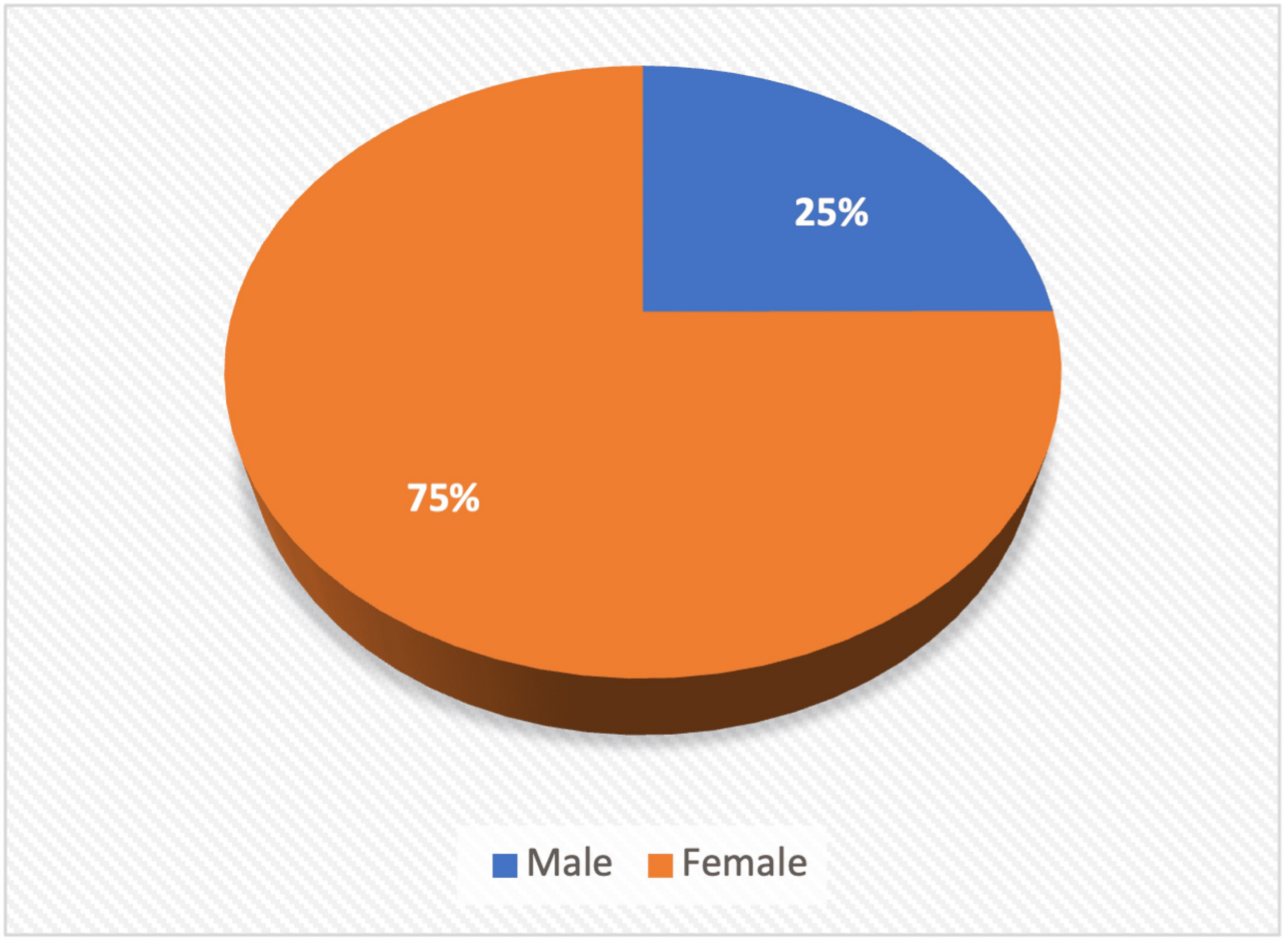
Pie chart showing distribution of sex of study population

The gender distribution showed a significant female predominance with a male-to-female ratio 1:3, as shown in Table 2. Figure 3, a pie chart, further emphasises this disparity, illustrating the higher risk and occurrence of hyperparathyroidism among females in our cohort.

**Table 2:** Gender distribution of the study population

Gender	Number	Percentage (%)
Male	1,498	25
Female	4,502	75
Total	6,000	100

Clinical presentations

**Figure 4:**
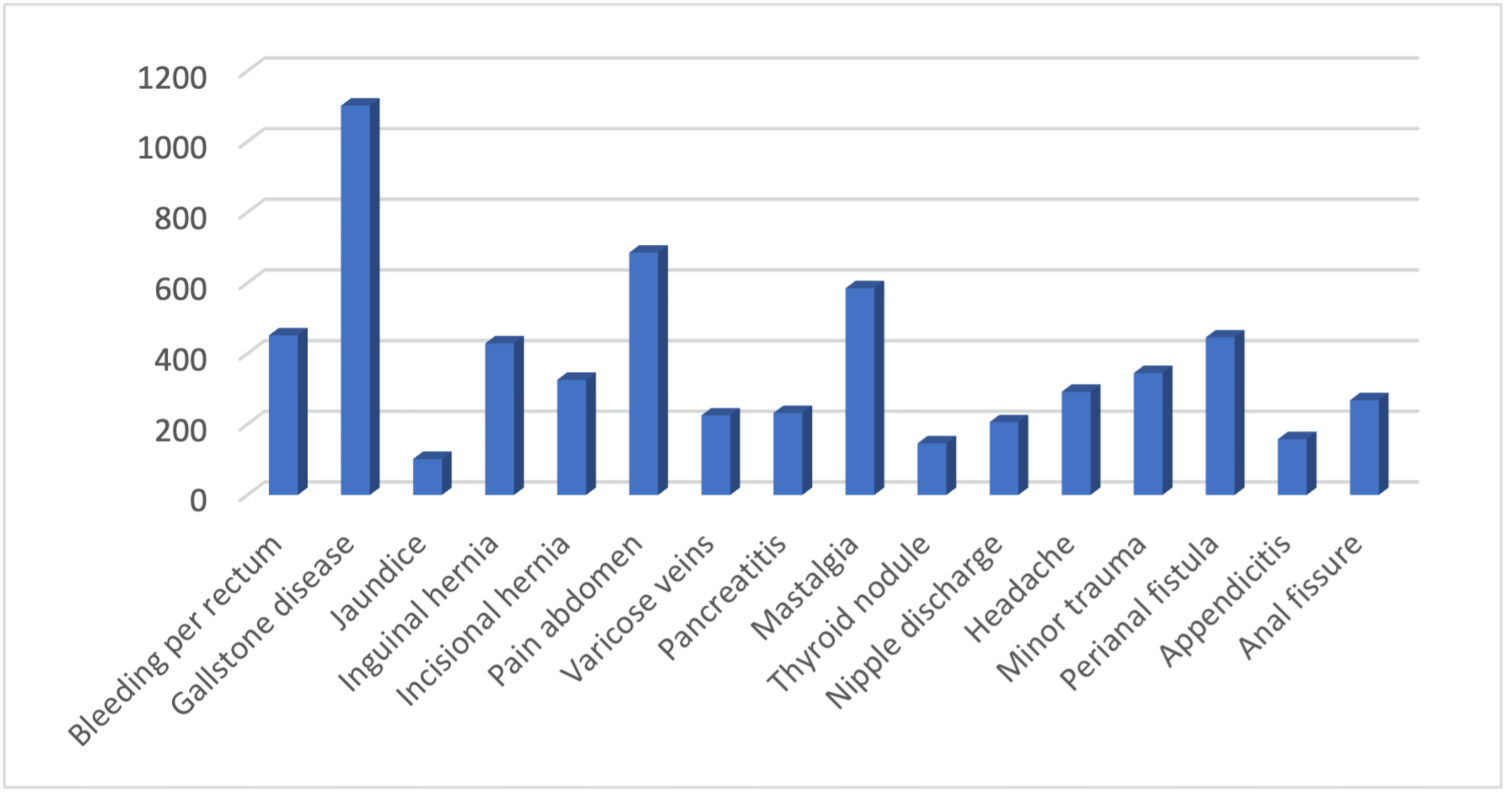
Bar chart showing various reasons for patients to attend surgery out-patient department

The primary reasons for attending the surgery outpatient department varied, with gallstone disease being the most common at 18.36%, followed by abdominal pain and bleeding per rectum, as summarised in Table 3. Figure 4 provides a bar chart visualising these various reasons, reflecting the variety of presentations that can coincide with undiagnosed metabolic abnormalities.

**Table 3:** Primary symptom/reason for attending the out-patient department

Symptom/Reason	Number	Percentage
Bleeding per rectum	452	7.52
Gallstone disease	1102	18.36
Jaundice	102	1.68
Inguinal hernia	429	7.12
Incisional hernia	326	5.4
Pain abdomen	686	11.44
Varicose veins	225	3.76
Pancreatitis	232	3.88
Mastalgia	585	9.76
Thyroid nodule	146	2.44
Nipple discharge	206	3.44
Headache	292	4.88
Minor trauma	345	5.76
Perianal fistula	446	7.44
Appendicitis	158	2.64
Anal fissure	268	4.48
Total	6000	100

Biochemical findings

**Figure 5:**
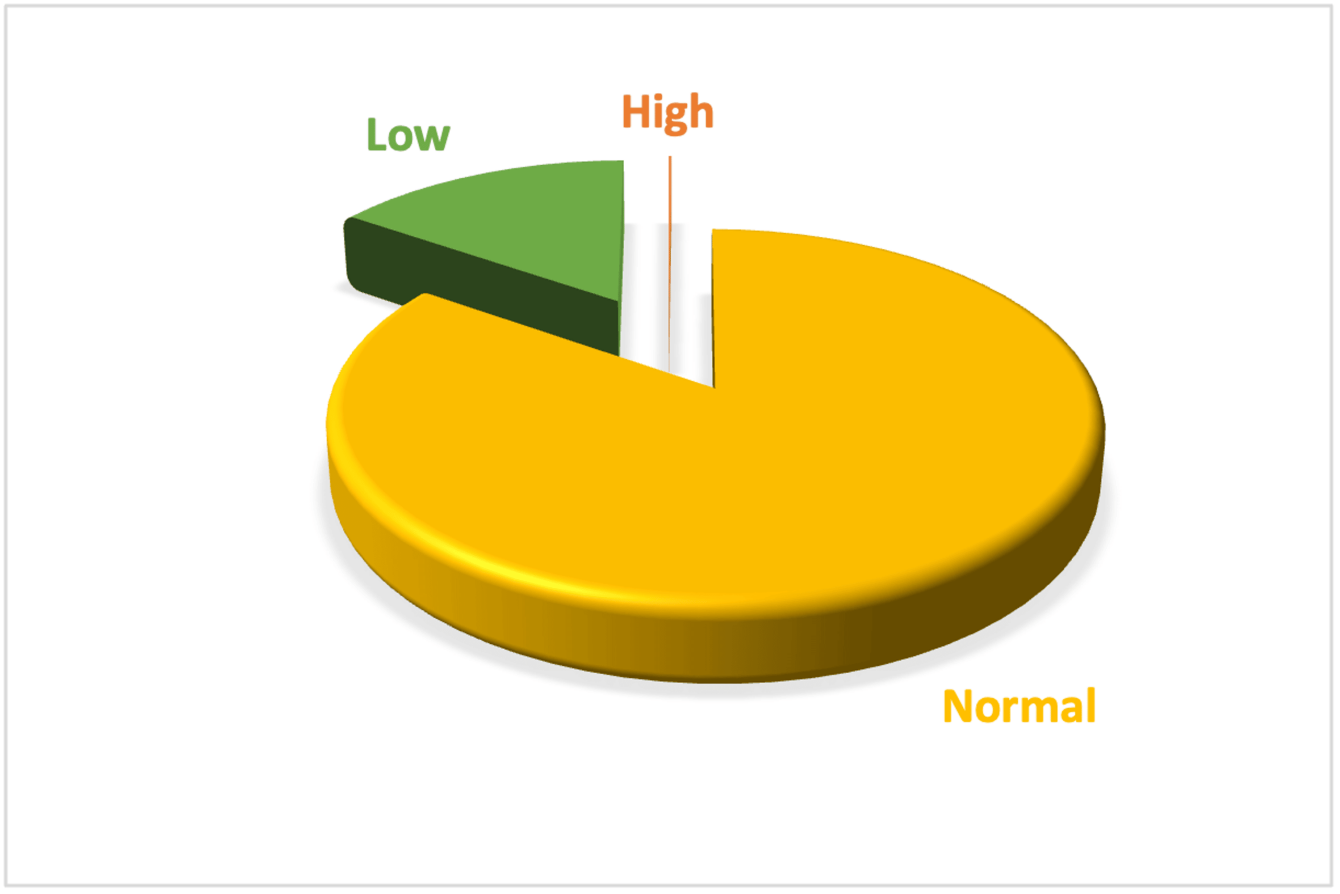
Pie chart showing the values of serum calcium in the study population

**Figure 6:**
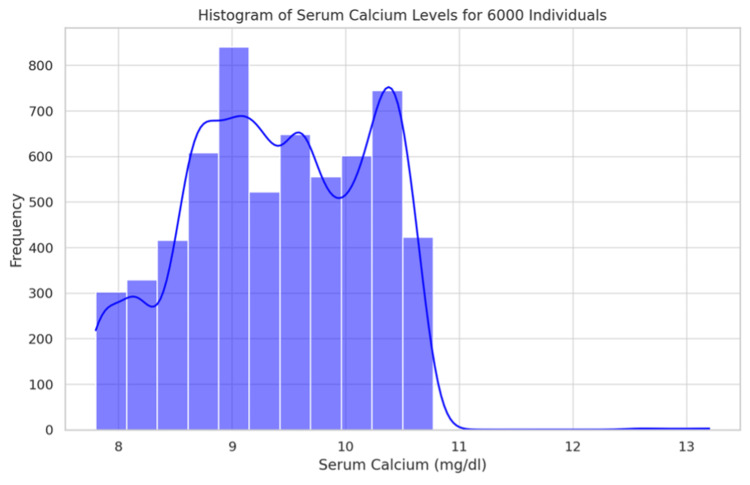
Histogram showing the distribution of serum calcium in the population

**Figure 7:**
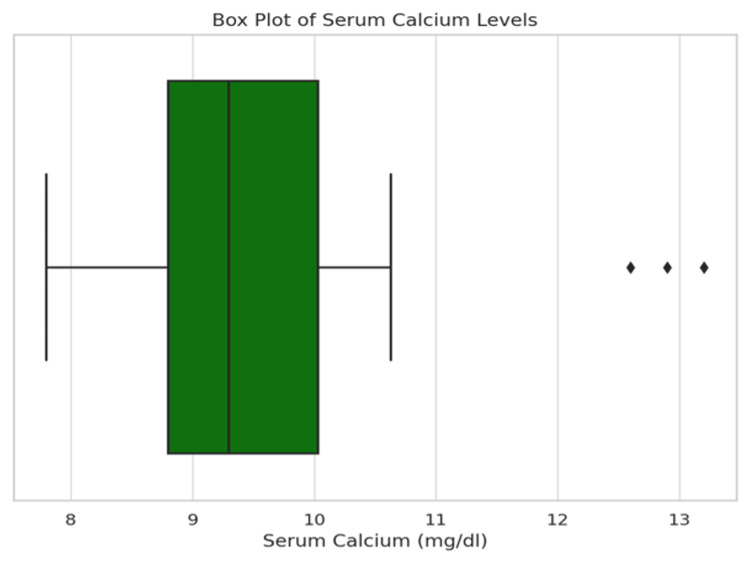
Box plot showing the distribution of serum calcium in the population

**Figure 8:**
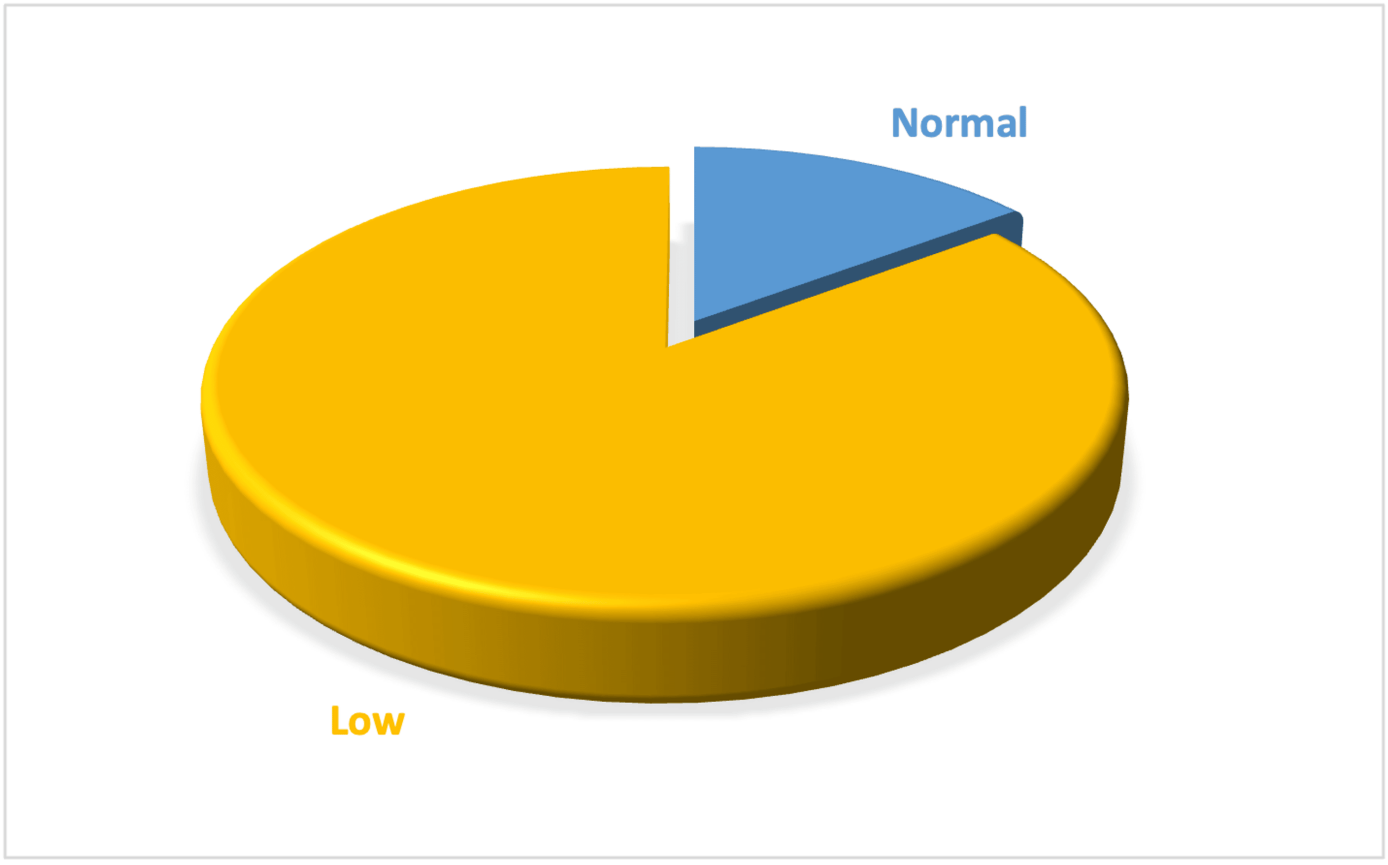
Pie chart showing the values of serum Vitamin D3 in the study population

The distribution of serum calcium is detailed in Table 4 and Figure 5, with 85.2% of participants displaying normal levels. The distribution is further analyzed in Figure 6 (Histogram) and Figure 7 (Box Plot), which delineate the overall distribution across the studied population. Table 5 and Figure 8 focus on Vitamin D3 levels, with 94.3% of the study population showing deficiency, a critical factor in calcium metabolism and a potential confounder in hyperparathyroidism detection.

**Table 4:** Values of serum calcium in the study population

Serum Calcium	Number	Percentage (%)
High	3	0.05
Normal	5,112	85.20
Low	885	14.75
Total	6,000	100

**Table 5:** Values of serum Vitamin D3 in the population

Serum Vitamin D3	Number	Percentage (%)
High	0	0
Normal	340	5.7
Low	5,660	94.3
Total	6,000	100

Among the study population, three patients were found to have high serum calcium and were included for further study. Three hundred thirty-seven patients had both normal serum calcium and normal serum Vitamin D3 and were excluded from further study. Five thousand six hundred and sixty patients had low serum Vitamin D3 and were administered Vitamin D3 supplementation, and serum calcium levels were then measured again in those patients. 

The impact of Vitamin D supplementation is explored in Table 6, where the majority of Vitamin D deficient individuals normalized their calcium levels post-supplementation. Table 7 further examines these changes, providing a detailed account of serum calcium adjustments after Vitamin D correction, indicating potential cases for further evaluation based on elevated levels post-correction.

**Table 6:** Combined values of serum calcium and Vitamin D3

Serum Calcium	Serum Vitamin D3	Number	Percentage (%)
High	Normal	3	0.05
Normal	Normal	337	5.62
Normal	Low	4,775	79.58
Low	Low	885	14.75
Total	Total	6,000	100

Effect of Vitamin D3 correction

Out of the 5660 patients with low serum Vitamin D3 who were administered Vitamin D3 supplementation, 575 patients with low serum calcium initially had an increase in the level of serum calcium to the normal range. Three hundred and ten patients with low initial serum calcium still had low levels of serum calcium after Vitamin D3 correction. Out of the 4775 patients with a normal serum calcium initially, two patients showed an increased level of serum calcium (higher than normal) after Vitamin D3 supplementation. Those two patients were included for further study. 

**Table 7:** Effect of Vitamin D3 correction on serum calcium values

Previous Serum Calcium	Serum Calcium After Correction	Number	Percentage (%)
Normal	Normal	4,773	84.30
Normal	High	2	0.04
Low	Normal	575	10.16
Low	Low	310	5.48
Total		5,660	100

Patient analysis

Out of the five patients, three had high serum iPTH values along with low serum phosphate, as depicted in Table 8. Gland localisation was done in them, followed by surgery. The other two patients had borderline high serum iPTH with low 24-hour urinary calcium levels.

**Table 8:** Detailed data of five patients for further study

Sl. No.	Previous Serum Calcium (mg/dl)	Serum Vitamin D3 (ng/ml)	Vitamin D3 Correction	Serum Calcium after Correction (mg/dl)	Serum Phosphate (mg/dl)	Serum iPTH (pg/ml)	24-hour Urinary Calcium (mg/day)
1	12.6	32	No	N/A	2.1	946	405
2	13.2	34	No	N/A	2.3	114	92
3	12.9	26	No	N/A	2.2	329	125
4	10.4	17	Yes	11.5	2.7	95	185
5	9.3	18	Yes	11.8	3.2	78	85

**Figure 9:**
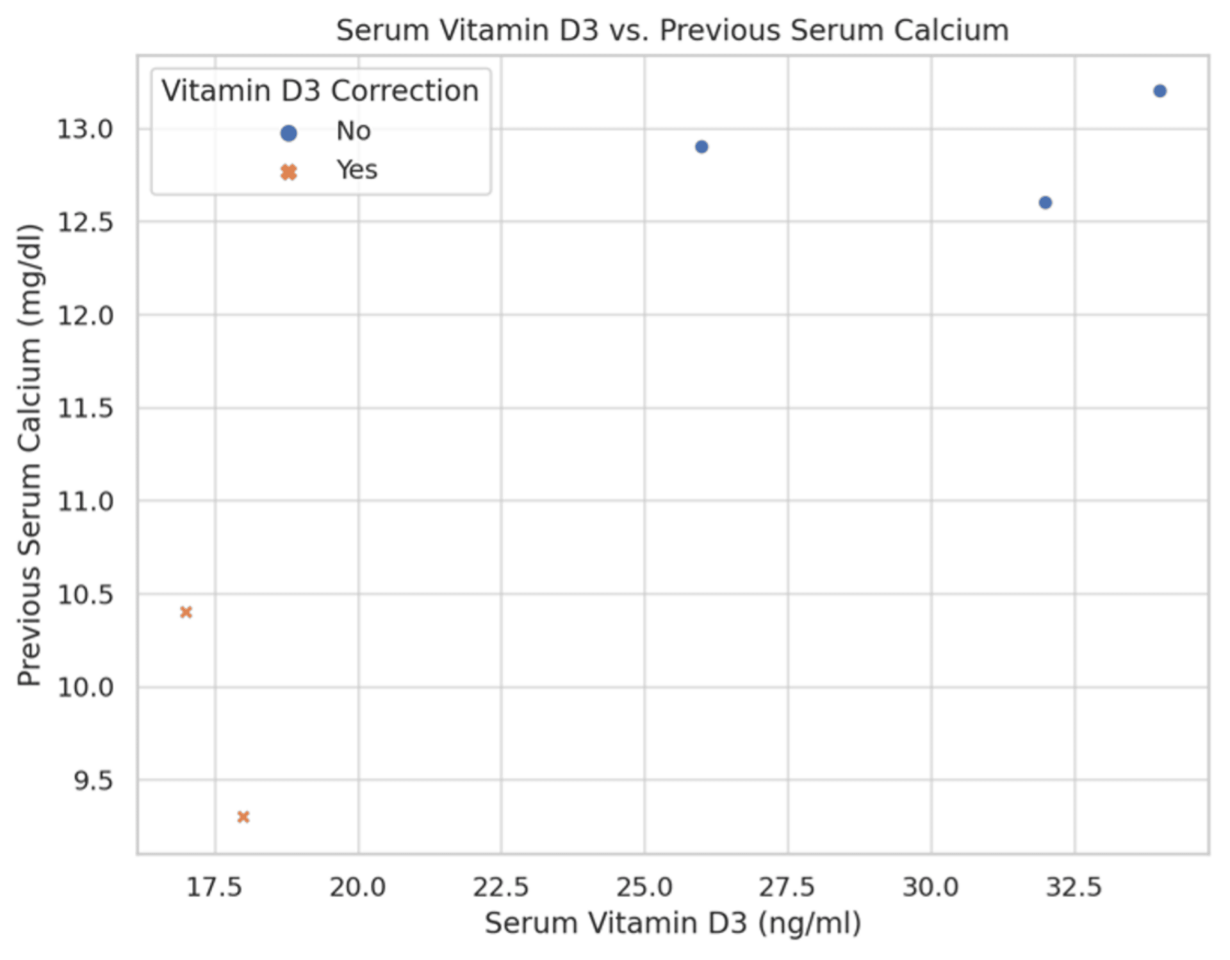
Scatter plot analysis of the relationship between previous serum calcium and Vitamin D3 levels A scatter plot showing the relationship between serum Vitamin D3 and previous serum calcium, including whether Vitamin D3 correction was applied.

**Figure 10:**
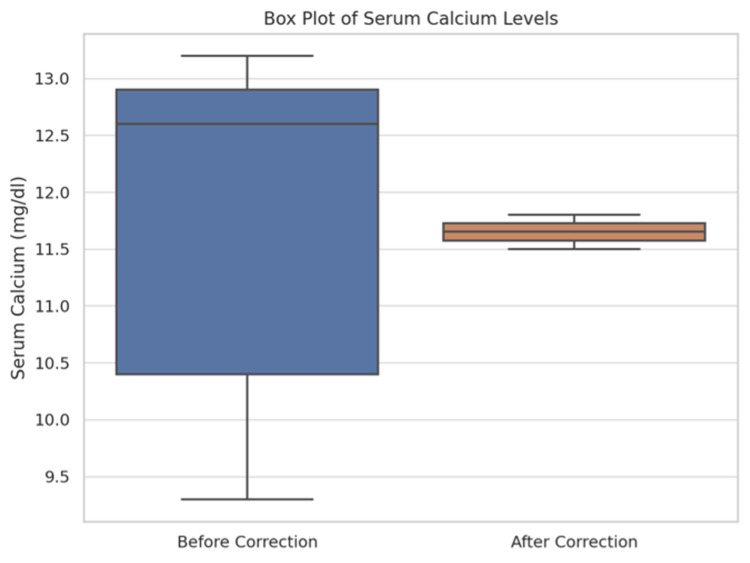
Box plot showing the distribution of serum calcium levels both before and after correction

Figure 9 offers a scatter plot analysis of the relationship between previous serum calcium and Vitamin D3 levels, including the effects of supplementation. Figure 10 presents a box plot of serum calcium levels before and after Vitamin D correction, visually summarising the biochemical impacts of the intervention. 

Table 9 summarises the outcomes of surgical intervention in patients diagnosed with primary hyperparathyroidism, indicating the effectiveness of the screening and subsequent surgical management in mitigating the effects of this condition. 

**Table 9:** Outcome of patients with primary hyperparathyroidism

Outcome	Number	Percentage (%)
Operated	16	84.21
Failed Localisation	2	10.53
Refused	1	5.26
Total	19	100

Statistical results

To evaluate the effectiveness of Vitamin D3 correction in managing serum calcium levels and to understand the demographic distribution of hypercalcemia, comprehensive statistical analyses were performed. These analyses were essential for validating the hypothesis that gender differences and Vitamin D3 correction status significantly influence serum calcium levels.

Descriptive statistics* *were utilised to summarise the basic features of the data, providing insights into the population distribution. This foundational analysis set the stage for more complex inferential statistical tests. A Chi-square test of independence was conducted to assess the association between gender and Vitamin D3 correction status. The aim was to determine if the distribution of Vitamin D3 correction was statistically different between male and female participants. The results revealed a Chi-square statistic of 1.63 with 1 degree of freedom, resulting in a p-value of 0.20. This indicates that there is no significant association between gender and Vitamin D3 correction status, suggesting that Vitamin D3 correction is uniformly applied across genders in the studied population.

An independent samples t-test was employed to compare the mean serum calcium levels before and after the administration of Vitamin D3. The analysis revealed a statistically significant difference in serum calcium levels post-correction (t(3778) = -7.715, p < 0.001). Prior to Vitamin D3 correction, the average serum calcium level was 9.38 mg/dl (SD = 0.79), which increased to 9.56 mg/dl (SD = 0.81) following correction. This significant increase supports the hypothesis that Vitamin D3 correction has a measurable effect on serum calcium levels, indicating that Vitamin D3 supplementation can effectively alter calcium homeostasis.

Logistic regression analysis was utilised to assess the factors influencing the emergence of hypercalcaemia post-Vitamin D correction. Among the initial screenings, only three cases of hypercalcemia were identified. However, post-Vitamin D correction, two additional cases of hypercalcaemia were detected, suggesting that Vitamin D supplementation may unmask underlying hypercalcaemia in certain patients. The model adjusted for age, gender, and baseline Vitamin D status, but found no significant predictors, indicating that the occurrence of hypercalcemia post-correction was random and not influenced by the demographic or baseline biochemical variables studied. These findings underscore the importance of assessing Vitamin D levels in patients with suspected disorders of calcium metabolism and provide a basis for further research into the optimal management of such conditions.

## Discussion

Out of the 6000 patients screened, 5112 (85.2%) had normal/low calcium levels with decreased Vitamin D3 levels and three had hypercalcemia with normal/low Vitamin D3. After Vitamin D3 correction in those 5112 patients, two more were found to have hypercalcemia. Serum phosphate and iPTH levels were then measured in those three plus two (five) patients. Out of five, three had high serum iPTH along with low serum phosphate. Gland localisation was done in them, followed by surgery. The other two patients had borderline iPTH values with low 24-hour urinary calcium levels. The prevalence of asymptomatic hyperparathyroidism in our study population was 0.05% (3/6000). During this period, we encountered 19 cases of PHPT (16 operated after localisation, one refused surgery, two had failure of localisation) out of which three were asymptomatic, 15.8% (3/19).

The literature states that the prevalence of asymptomatic hyperparathyroidism in the population is 1% globally and 3% in India in the first decade of the century, and around 13% in the second decade of the century. This shift has been assigned to the fact that the there have been more number of tests for serum calcium and serum Vitamin D3 in the second decade of the century. As per reports, 5% of patients with PHPT are asymptomatic in India. 

In our study, we found the overall incidence of asymptomatic hyperparathyroidism to be 0.05% and 15.8% of our patients of PHPT were asymptomatic. The observed prevalence of asymptomatic hyperparathyroidism (0.05%) in this study is significantly lower than global estimates (1%) and even Indian reports (3-5%). This discrepancy may reflect limited routine screening practices and Vitamin D deficiency, which masks hypercalcemia. The study underscores the need for targeted screening in high-risk groups to ensure early detection and intervention. Comparative analysis of findings with global and regional data highlights the potential influence of Vitamin D deficiency and socio-economic factors on PHPT prevalence. Despite its rarity, early detection of asymptomatic cases is crucial to mitigate long-term complications and reduce the healthcare burden associated with advanced disease.

Review of literature

Primary hyperparathyroidism (PHPT) is a relatively common endocrine disorder with significant variations in clinical presentation and prevalence worldwide. Historically, severe symptoms such as osteitis fibrosa cystica and kidney stones were predominant before the 1970s, as described by Sivula and Ronni-Sivula [17]. The advent of automated calcium measurement has since facilitated the detection of asymptomatic and minimally symptomatic cases, as reported by Heath et al. [18].

Global Prevalence and Trends

Wermers et al. [28] conducted a population-based study in Rochester, Minnesota, highlighting a declining incidence of symptomatic PHPT cases alongside a rise in mild and asymptomatic presentations. These findings were echoed by Lundgren et al. [19], who noted that over 80% of PHPT patients in Western countries now present without classic severe symptoms. The global prevalence of asymptomatic PHPT is estimated at approximately 1% among adults, with a higher incidence in postmenopausal women.

Indian Context

In contrast, studies in India reveal a higher prevalence of symptomatic PHPT, with limited data on asymptomatic cases. Bhansali et al. [33] and Maskey et al. [34] reported that most Indian patients present with bone disease, nephrolithiasis, or both, often attributed to delayed diagnosis and widespread Vitamin D deficiency. Pradeep et al. conducted a systematic review of Indian studies, emphasizing the significant delay in seeking medical attention compared to developed nations [35]. Mithal et al. [36] identified asymptomatic PHPT in 38% of cases in North India, a notable finding given the traditionally symptomatic presentation in the region.

Diagnostic Challenges and Advances

The shift from severe to mild and asymptomatic presentations globally raises questions about diagnostic thresholds and management strategies. Silverberg et al. [9] and Rubin et al. [8] demonstrated that many asymptomatic patients remain stable without intervention. However, surgical management remains the definitive treatment for those meeting established guidelines, as outlined in the National Institute of Health (NIH) Consensus Statement [3].

Gaps and Implications

Despite advances, PHPT remains underdiagnosed in regions like India, where routine screening is not widespread. The interplay between Vitamin D deficiency and hypercalcemia further complicates diagnosis, as highlighted by Maskey et al. [34]. This underscores the need for targeted screening programs, particularly in high-risk populations, to identify asymptomatic cases early and reduce morbidity.

Limitations

One of the main limitations of this study is its potential selection bias due to the specific setting of a tertiary care center, which may not accurately represent the general population. Patients attending such centers could have differing baseline characteristics compared to a more diverse community sample, possibly affecting the generalizability of the findings. Additionally, the study's design as a single-point screening without subsequent long-term follow-up means that the long-term outcomes of the diagnosed patients, particularly the progression of the disease and response to surgery, remain unknown. These outcomes are crucial for understanding the true benefit of early detection and intervention in asymptomatic hyperparathyroidism.

The diagnostic criteria, relying primarily on biochemical markers, may also limit the detection of patients with mild or nonspecific symptoms that could otherwise suggest hyperparathyroidism. Furthermore, the exclusion of individuals with conditions that could affect calcium metabolism, while reducing confounding, might also exclude a subset of patients who could have provided deeper insights into the complexities and spectrum of the disease. The other critical aspect is the study's statistical power, which might not be sufficient to detect smaller but clinically meaningful differences or to conduct comprehensive subgroup analyses due to the very low event rate of confirmed asymptomatic hyperparathyroidism.

To summarise, this study has several limitations that should be acknowledged. First, it was conducted at a single tertiary care centre, which may limit the generalisability of the findings. Second, the retrospective design carries inherent biases, including reliance on the accuracy of existing records. Lastly, while efforts were made to ensure data completeness, a formal analysis of missing data was not performed, which may influence the robustness of some results. Future multicentre, prospective studies with standardised data collection would help validate and extend these findings.

Implications for future research

Future studies should consider a multi-center design to include a broader geographic area and a more diverse population to enhance the external validity of the findings. Long-term follow-up of screened individuals would be valuable to assess the progression of asymptomatic hyperparathyroidism and the long-term benefits of surgical versus conservative management, especially in terms of bone mineral density, kidney function, and overall quality of life.

Moreover, integrating additional diagnostic tools such as neck ultrasound or more sensitive biomarkers could improve the detection rates and help in better stratification of patients who may benefit from early intervention. It would also be beneficial to explore the impact of Vitamin D correction in more detail, assessing how it influences serum calcium levels and potentially masks or modifies the course of hyperparathyroidism.

## Conclusions

This study demonstrates the feasibility of using serum calcium and Vitamin D screening to identify asymptomatic hyperparathyroidism in a tertiary care population. Although the prevalence is lower than global estimates, early diagnosis and surgical intervention in confirmed cases can significantly reduce morbidity and healthcare costs. Routine screening should be considered, particularly in regions with high rates of Vitamin D deficiency.
